# Supplementary material for: Asynchronous magnetic resonance elastography: Shear wave speed reconstruction using noise correlation of incoherent waves
Source: Magn Reson Med. 2022 Oct 27;89(3):990–1001. doi: 10.1002/mrm.29502 (PMC9792433; doi:10.1002/mrm.29502)
Supplement: Supplementary file 1 — DATA S1 MATLAB code used to generate the numerical simulations of Figure 1 [file MRM-89-990-s002.zip › k-Wave/helpfiles/example_pr_2D_tr_absorption_compensation.html]

Attenuation Compensation Using Time Reversal Example (k-Wave)


# Attenuation Compensation Using Time Reversal Example

This example demonstrates how the acoustic attenuation present in the photoacoustic forward problem can be compensated for using time reversal image reconstruction. It builds on the 2D Time Reversal Reconstruction For A Circular Sensor Example.

- Open the file in the MATLAB Editor
- Run the file in MATLAB

For a more detailed discussion of this example and the underlying techniques, see B. E. Treeby, E. Z. Zhang, and B. T. Cox, "Photoacoustic tomography in absorbing acoustic media using time reversal," *Inverse Problems*, vol. 26, no. 11, p. 115003, 2010.

## Contents

- Running the forward simulation
- Selecting the regularisation parameters
- Time reversal image reconstruction

## Running the forward simulation

The sensor data is simulated using `kspaceFirstOrder2D` in a similar manner to previous examples. The initial pressure is set to the Shepp Logan phantom and a circular Cartesian sensor mask with 200 sensor points is used to record the data. After the simulation is complete, random Gaussian noise is added using `addNoise` to give a signal to noise ratio of 40dB (based on the peak of the recorded signal and the root-mean-squared noise level).

```
% run the forward simulation
sensor_data = kspaceFirstOrder2D(kgrid, medium, source, sensor, input_args{:});

% add noise to the recorded sensor data
signal_to_noise_ratio = 40;	% [dB]
sensor_data = addNoise(sensor_data, signal_to_noise_ratio, 'peak');
```

## Selecting the regularisation parameters

To compensate for the acoustic attenuation in the forward problem, the absorption parameter must be reversed in sign (the frequency content must grow rather than decay). This is achieved by setting `medium.alpha_sign` to `[-1, 1]`. The two inputs control the sign of the absorption and dispersion parameters, respectively. The dispersion parameter (which controls the dependence of the sound speed on frequency) should not be reversed because if the high frequency components of the wave field have travelled to the detector faster than the low frequency components (as is the case for photoacoustic signals in biological tissue), they again need to travel faster than the low frequency components in the time reversal reconstruction to regain their initial position within the medium.

If a photoacoustic image is reconstructed from real or noisy data with the absorption parameter reversed, the high frequency content (where the signal to noise ratio is typically quite low) can quickly grow to mask the low frequency content, effectively obscuring the desired features within the reconstructed image. This is because, as the waves propagate, the high frequencies are increased at a much faster rate (recall the acoustic attenuation in soft biological tissue follows a frequency power law). To avoid this, the absorption and dispersion parameters are filtered using a frequency domain Tukey window. This regularises the reconstruction (in effect, stops it from 'blowing up') by restricting the range of frequencies that are allowed to grow. The filter is applied to the absorption parameters by assigning it to `medium.alpha_filter`. The filter must have the same number of dimensions and grid points as `kgrid.k`.

To choose an appropriate filter cutoff frequency, the average power spectrum of the simulated sensor data is computed. This is plotted below (shown in black), along with the power spectrum of the signal recorded at the first sensor point (shown in red). In this example, the filter cutoff frequency (shown as the left dashed line in the figure) is chosen based on the noise floor observable in the power spectrum. The maximum frequency supported by the grid is also shown (right dashed line in the figure).

Here, a symmetric Tukey window (or tapered cosine window) scaled to a particular frequency cutoff is used so that the correct power law absorption and dispersion characteristics are maintained within the filter pass band. The filter is created using `getAlphaFilter` which creates the filter using `getWin` via rotation.

```
% define the cutoff frequency for the filter
f_cutoff = 3e6;                     % [Hz]

% create the filter to regularise the absorption parameters
medium.alpha_filter = getAlphaFilter(kgrid_recon, medium, f_cutoff);

% reverse the sign of the absorption proportionality coefficient
medium.alpha_sign = [-1, 1];        % [absorption, dispersion];
```

A visualisation of the filter is given below.


## Time reversal image reconstruction

The initial pressure distribution, and the reconstructions with and without compensation for acoustic attenuation are shown below. Profiles through x = 0 are also shown for comparison. Without correction for attenuation, the edges of the reconstructed pressure become blurred, and the overall magnitude is reduced. When compensation for acoustic attenuation is included into the reconstruction, the sharpness and magnitude of the reconstructed pressure is considerably improved. Due to the band-limited frequency response of the sensors (high frequencies are not detected because their magnitudes are below the noise floor of the added noise), the reconstruction is unable to completely recover the frequency content of the initial pressure distribution. This results in Gibbs phenomenon appearing in the reconstruction where there are sharp changes in the initial pressure.
